# Vitamin D Status in Russian Children and Adolescents: Contribution of Genetic and Exogenous Factors

**DOI:** 10.3389/fped.2020.583206

**Published:** 2020-11-19

**Authors:** Elena I. Kondratyeva, Irina N. Zakharova, Natalya A. Ilenkova, Leonid Ya. Klimov, Nika V. Petrova, Aisa E. Zodbinova, Elena K. Zhekaite, Vladimir V. Chikunov, Svetlana V. Dolbnya, Anna Yu. Voronkova, Victoria D. Sherman, Elena V. Loshkova, Yuliya L. Melyanovskaya, Roman M. Budzinskiy, Victoria A. Kuryaninova, Sergey I. Kutsev

**Affiliations:** ^1^Research Centre for Medical Genetics, Moscow, Russia; ^2^Department of Paediatrics, Russian Medical Academy of Continuous Postgraduate Education, Moscow, Russia; ^3^Voino-Yasenetsky Krasnoyarsk State Medical University, Krasnoyarsk, Russia; ^4^Department of Paediatrics, Stavropol State Medical University, Stavropol, Russia

**Keywords:** vitamin D deficiency, polymorfism, age features, insolation, VDR

## Abstract

**Background:** The problem of vitamin D deficiency is particularly relevant for the entire territory of Russia, since most parts of the country are located above the 42nd geographical latitude and the residents are therefore at risk of vitamin D deficiency. Despite the urgency of the problem, a comprehensive study of the molecular and genetic mechanisms and exogenous factors of vitamin D deficiency in children living in various geographical areas of the Russian Federation has not been conducted. Different variants in the loci of the genes responsible for the synthesis, hydroxylation, and transport of vitamin D (such as *DHCR7, CYP2R1, CYP24A1*, and *GC*), as well as *VDR* gene polymorphisms may also be associated with the risk of vitamin D deficiency. The aim of this study was to analyze the influence of exogenous factors on the blood levels of 25-hydroxyvitamin D (25(OH)D) in children of three regions of the Russian Federation, as well as the relationship of blood 25(OH)D levels with polymorphic variants of cytochrome P450 genes and *VDR* gene.

**Methods:** We conducted blood 25(OH)D level analysis in 333 healthy children and adolescents in three regions located in different geographical zones of the Russian Federation. We studied the polymorphic variants c.1075A>C (I359L, rs1057910, CYP2C9^*^3) and c.430C>T (R144C, rs1799853, CYP2C9^*^2) in the *CYP2C9* gene, c.1334T>C (M445T, rs4986910, CYP3A4^*^3), and CYP3A4^*^1B (c.-392C>T, rs2740574) in the *CYP3A4* gene, 1846G>A, (rs3892097, CYP2D6^*^4) in the *CYP2D6*gene, *Taq*I (NM_000376.2: c.1056T>C; rs731236), *Fok*I (NM_000376.2:c.2T>C; (rs2228570), and *Bsm*I (NM_000376.2: c.1024+283G>A; rs1544410) in the *VDR* gene. We also analyzed the influence of exogenous factors on the level of 25(OH)D in children of the three study regions, as well as the relationship of the level of 25(OH)D with variants CYP2C9^*^2 (c.430C>T; R144C), CYP2C9^*^3 (c,1075A>C; I359L), CYP2D6^*^4 (1846G>A), CYP3A4^*^3 (c.1334T>C), and CYP3A4^*^1B (c.-392C>T) and rs731236, rs2228570 and rs1544410 in the *VDR* gene.

**Results:** We found that the blood level of 25(OH)D depended on the geographical location and the number of sunny days per year. The average blood level of 25(OH)D in adolescent boys was statistically significantly lower than in girls of this age group. The level of 25(OH)D also significantly depended on the prophylactic dose of cholecalciferol administered to the subjects. In the study, it was shown that a dose of cholecalciferol ≥1,000 IU per day can achieve a normal level of 25(OH)D in healthy children. We found no statistically significant association between single-nucleotide polymorphic variants of cytochrome P450 genes (CYP2C9^*^3, CYP3A4^*^3, CYP2C9^*^2, CYP2D6^*^4, and CYP3A4^*^1B) and blood level of 25(OH)D in the subjects. We also did not find a relationship between the *Taq*I, *Fok*I, and *Bsm*I polymorphisms of the *VDR* gene and serum 25(OH)D concentration.

**Conclusion:** Exogenous factors (time of year, place of residence, and prophylactic administration of cholecalciferol), as well as endogenous factors (age and sex), play a determining role in the development of vitamin D deficiency and insufficiency; in contrast to genetic factors—polymorphic variants of the genes of xenobiotic phase 1 enzymes (*CYP2C9, CYP2C19, CYP2D6*, and *CYP3A4*) and the *VDR* gene—which do not play such role. This study shows the need to create a diagnostic algorithm for Vitamin D deficiency based on the age, season of the year, and prophylactic dose of cholecalciferol.

## Introduction

Vitamin D, which belongs to the group of fat-soluble vitamins, plays important roles in the human body. Its main role is in the metabolism of bone tissue—maintaining mineralization, bone growth, and the process of bone remodeling. However, the functions of vitamin D are not limited to the regulation of calcium-phosphorus metabolism: it also affects the processes of cell proliferation and differentiation, neuromuscular impulse transmition, and modulation of immune response and inflammation (1, 2). Important for Vitamin D synthesis in the body is the presence of ultraviolet rays of sunlight and their contact with the skin (3–5). Vitamin D, obtained from food and in the form of food additives, as well as formed during exposure to the sun is biologically inert. To be converted into an active form−1,25(OH)2D, it must undergo two processes of hydroxylation in the body. At the first stage of hydroxylation in the liver by the action of 25-hydroxylase (CYP2R1), 25-hydroxyvitamin D [25(OH)D, also called calcidiol] is formed. While the second hydroxylation in the kidneys by the action of CYP27B1 (1α-hydroxylase), leads to formation of the biologically active form, 1,25(OH)2D (6). In enzyme identification studies, cytochrome P450 isoforms—CYP2C9, CYP3A4, and CYP2D6—have been shown to participate in vitamin D hydroxylation reactions (7, 8). It was also found that 1,25(OH)2D induces the expression of the *CYP3A4, CYP2B6*, and *CYP2C9* genes in primary human hepatocytes, and vitamin D receptors (VDRs) are able to bind and transactivate various motifs recognized by xenobiotics via the X-pregnan receptor and constitutive androstane receptors in the promoter regions of these cytochrome genes (9).

Due to the low content of vitamin D in most foods, the main source of this vitamin is its formation under the influence of ultraviolet rays. Recently, due to reduction in sunlight, vitamin D deficiency has become very common in both adults and children (10). The problem of vitamin D deficiency is particularly relevant in the entire territory of Russia, since most parts of the country are located above the 42nd geographical latitude, and the residents are thus at risk of vitamin D deficiency (11). However, no research has been conducted about vitamin D deficiency for different regions and different seasons of the year in Russia.

Different variants in the loci of genes (such as DHCR7, CYP2R1, CYP24A1, and GC) that are responsible for vitamin D synthesis, hydroxylation, and transport, as well as VDR gene polymorphisms, may be associated with the risk of vitamin D deficiency (12, 13). Most tissues and cells in the body contain VDR. Following the genomic pathway in cells the active form of vitamin D binds to VDR and forms a complex. In the nucleus, this complex heterodimerizes with the retinoic acid x-receptor and affects the transcription of vitamin D-dependent genes. VDR is a nuclear transcription factor and has similarities to steroid and thyroid hormone receptors. It is encoded by the *VDR* gene located on chromosome 12 at position 13.11 (12q13.11).

The *VDR* gene has 11 exons (8 of them encoding) and contains approximately 75 kb. Several polymorphic variants of the *VDR* gene have been identified, but the most studied are four single-nucleotide polymorphic variants, which are traditionally designated by the names of the restriction endonucleases with which they were detected: BsmI, ApaI, TaqI, and FokI (14). The TaqI (NM_000376.2: c.1056T>C; rs731236) polymorphism is located in exon 11 (8th encoding exon) near the 3' end of the *VDR* gene and results in a synonymous change in p. Ile352 due to the replacement of c. 1056T>C. The FokI polymorphism (rs2228570, NM_000376.2:c.2T>C; NP_000367.1:p.Met1Thr) is located in the 4th exon of the gene (1st encoding exon), and its presence leads to a change in the starting codon. The *VDR* BsmI (rs1544410, NM_000376.2: c.1024+283G>A) and ApaI (rs7975232, NM_000376.2:c.1025-49G>T) *VDR* polymorphisms are located in intron 10 at the 3' end of the gene: these polymorphisms do not alter the amino acid sequence of the VDR protein, but they can affect gene expression through changes in mRNA stability, disruption of splicing sites for mRNA transcription, or changes in intron regulatory elements. Studies have established the association of *VDR* gene polymorphism with diseases such as osteoporosis, diabetes, tuberculosis, hyperparathyroidism, chronic renal failure, and various neoplasms, as well as various cardiovascular diseases (14–16). A relationship between vitamin D levels and certain diseases has also been found (17, 18). Despite this, no previous study on the prevalence of vitamin D deficiency in children in the Russian Federation including a comprehensive approach to assessing both genetic factors and exogenous factors has been done.

The purpose of this study was to analyze the influence of exogenous factors on the blood levels of 25(OH)D in children of three regions of the Russian Federation, as well as the relationship of blood 25(OH)D levels with polymorphic variants of CYP2C9^*^2 (c.430C>T; R144C), CYP2C9^*^3 (c.1075A>C; I359L), CYP2D6^*^4 (1846G>A), *CYP3A4* (c.1334T>C), and CYP3A4^*^1B (c.-392C>T) and BsmI, TaqI, and FokI polymorphisms of the *VDR* gene.

## Materials and Methods

### Study Subjects

This prospective, non-randomized, multicenter, cohort study included healthy children and adolescents aged 0–18 years (*n* = 333): 145 subjects were from the Moscow region (64 boys and 81 girls; average age, 7.1 ± 4.7 years; median age, 6.3 years), 137 subjects were from the Krasnoyarsk territory (62 boys and 75 girls; average age, 7.0 ± 5.2 years; median age, 6.7 years), and 51 subjects were from the Stavropol territory (21 boys and 30 girls; average age, 8.1 ± 4.8 years; median age, 9.0 years). The subjects were examined in the four seasons of the year (Table 1).

**Table 1:** Number of samples and studies conducted in healthy subjects in different seasons of the year.

**Region**	**Samples (*n*) winter**	**Samples (*n*) spring**	**Samples (*n*) summer**	**Samples (*n*) autumn**	**Samples (*n*) total**
Moscow	71	12	22	46	151
Krasnoyarsk	36	32	35	34	137
Stavropol	49	29	30	49	157
Total	156	73	87	129	445

The inclusion criteria were absence of chronic diseases and acute respiratory infections 1 month before blood collection and signing of informed consent by parents/guardians. Children were included in the study after informed consent was obtained.

In the analysis, subjects were divided into three age groups (Table 2). Children were classified into the 0–3 years group, 4–10 years group, and 11–18 years group, in accordance with the periodization of children's ages by the Russian Federation (19).

**Table 2:** Average level of 25(OH) D (ng/ml) in subjects, depending on age.

**Age, years**	**M ± m**	**Me(Q1–Q3)**	***p***
0–3 (1)	41.36 ± 24.48	35.85 (24.40–50.10)	*p*_1−2_ = 0.002
4–10 (2)	32.37 ± 13.92	31.12 (22.82–38.80)	
11–18 (3)	26.94 ± 12.49	24.60 (18.30–31.55)	*p*_1−3_ <0.001
Total	32.74 ± 17.47	29.60 (21.68–39.70)	*p*_2−3_ <0.001

*p is the level of significance of differences, and Mann–Whitney test was applied*.

The study was approved by the EC of the Research Center for Medical Genetics, Protocol “Algorithm for diagnosis and correction of vitamin D deficiency in children of the Russian Federation” No. 9, dated December 8, 2017.

### 25(OH)D Assay

All biochemical and genetic analyses were performed at the Research Center for Medical Genetics laboratory.

The blood samples were collected at the children's outpatient department (Moscow, Krasnoyarsk, Stavropol) and were transported and stored at 25°C. All samples were taken simultaneously at each season to determine the level of 25(OH)D. The analyses as performed using the same equipment by the same laboratory assistants.

The blood level of vitamin D was assessed by assaying its intermediate metabolite, 25(OH)D. Enzyme immunoassays were performed with EUROIMMUN AG kits (Germany) using the EnSpire flatbed spectrofluorometer (PerkinElmer, Finland), Biosan Laboratory Centrifuge LMC-3000, and Biosan Thermo-Shaker PST-60-HL-4.

Results of the 25(OH)D assay were interpreted in accordance with the recommendations of the International Society of Endocrinologists (2011): severe deficiency, 25(OH)D level <10 ng/ml; deficiency, 10–20 ng/ml; insufficiency, 21–29 ng/ml; and normal level, 30–100 ng/ml. Levels more than 100 ng/ml were considered excessive and required adjustment of the dose of vitamin D (20).

Data on the duration of sunshine (DS) (the number of solar hours per month during the years of study) were obtained from the archives of the meteorological service for each region (Table 3).

**Table 3:** Duration of sunshine (hours) in the three study regions in 2018.

**Regions**	**Months**											
	**I**	**II**	**III**	**IV**	**V**	**VI**	**VII**	**VIII**	**IX**	**X**	**XI**	**Total**
Moscow	20.9	60.8	161.2	240.4	340.6	324.8	206.3	352.7	208.9	115.7	67.1	2099.4
Krasnoyarsk	62.8	112.8	180.3	197.1	183.9	341.5	254.4	300.7	153.5	144.5	47.2	1,978.7
Stavropol	38.3	49.1	92.5	304.3	290.2	366.0	301.2	346.1	223.9	243.6	91.8	2347.0

### Molecular and Genetic Analysis

DNA was extracted from peripheral blood leukocytes using a commercial set of DNA extraction reagents manufactured by Wizard® (Genomic DNA Purification Kit).

The analysis of polymorphic variants of cytochrome and *VDR* genes was performed by polymerase chain reaction, followed by restriction with specific endonucleases. Polymorphisms of CYP2C9^*^3 (c.1075A>C; I359L, rs1057910), CYP3A4^*^3 (c.1334T>C; M445T, rs4986910), CYP2C9^*^2 (c.430C>T; R144C, rs1799853), CYP2D6^*^4 (1846G>A, rs3892097), and CYP3A4^*^1B (c.−2212392C>T, rs2740574), and polymorphic variants of the *VDR* gene— NM_000376.2: c.1056T>C (rs731236), NM_000376.2:c.2T>C (rs2228570), NM_000376.2: c.1024+283G>A (rs1544410)—were studied. The used primers, restriction endonucleases, the length of amplified fragments and the length of restriction products are shown in Table 4.

**Table 4:** Primer sequences and restriction endonucleases for determining polymorphic variants of cytochrome and *VDR* genes.

**Gen**	**Variant**	**Primer sequence**	**Amplified fragment length (bp)**	**Resriction endonuclease**	**Products of restriction (bp)**
*CYP2C9*	CYP2C9*3 (1075A>C; I359L)	F 5′ TGCACGAGGTCCAGAGATAT R 5′ACCCGGTGATGGTAGAGGTT	183	EcoRV	1075A−183; 1075C−165+18
*CYP3A4*	CYP3A4*3 (c.1334T>C; M445T)	F 5′ GGACACATCACCACCCTGAAT R 5′ TGTTCAGGAGAGCAAACCTC	395	FaeI	1334T−233+139+23; 1334C−233+162
*CYP2C9*	CYP2C9*2 (c.430C>T; R144C)	F 5′ GCATGTGCCTGTTTCAGCAT R 5′ TATGGCCACCCCTGAAATGT	400	AspS9I	430C−81+177+142; 430T−81+319
*CYP2D6*	CYP2D6*4 (c.1846G>A)	F 5′ AGAAGGGCACAAAGCGGGAA R 5′ AGAGACTCCTCGGTCTCTCGC	264	MvaI	1846G−73+191; 1846A−264
*CYP3A4*	CYP3A4 *1B (c.-392C>T)	F 5′CAGCCATAGAGACAAGGGCC R 5′ACACACACCACTCACTGACC	173	MspI	−392C−19+154; −392T−173
*VDR*	c.2T>C (FokI)	F 5′ GCCAGCTATGTAGGGCGAAT R 5′ AGGAGGGCTCACCTGAAGAA	368	FokI	2T−131+28+63+146; 2C−131+28+209
*VDR*	c.1056T>C (TaqI)	F 5′ CTGAGAGCTCCTGTGCCTTC R 5′ ACAAGGGGCGTTAGCTTCAT	271	TaqI	1056T−271; 1056C−73+198
*VDR*	c.1024+283G>A (BsmI)	F 5′ CCTGAAGGGAGACGTAGCAA R 5′ CACTGCACATTGCCTCCAAA	351	BsmlI	1024+283G−198+153; 1024+283A−351

The genotypes of the variants studied were in Hardy-Weinberg Equilibrium (Table 5).

**Table 5:** Frequencies of genotypes and alleles of polymorphic variants of *CYP* and *VDR* genes.

**Polymorphic variants**	**Genotype (allele)**	***n***	**%**	**χ2(1df)**	***p***
CYP2C9 (1075A>C; I359L)	AA	106	88.33	0.4605	>0.05
	AC	14	11.67		
	CC	0			
	A	226	94.17		
	C	14	5.83		
CYP3A4 (c.1334T>C; M445T)	TT	94	100.00	–	–
	TC	0			
	CC	0			
	T	188	100.00		
	C	0			
CYP2C9 (c.430C>T; R144C)	CC	98	81.67	1.2221	>0.05
	CT	22	18.33		
	TT	0			
	C	218	90.83		
	T	22	9.17		
CYP2D6 (1846G>A)	GG	79	67.52	2.5313	>0.05
	GA	31	26.49		
	AA	7	5.99		
	G	189	80.77		
	A	45	19.23		
CYP3A4 (c.−392C>T)	CC	0	0	0.0796	>0.05
	CT	6	5.04		
	TT	113	94.96		
	C	6	2.52		
	T	232	97.48		
VDR (c.1056T>C; TaqI)	TT	74	47.74	0.6560	>0.05
	TC	63	40.65		
	CC	18	11.61		
	T	211	68.06		
	C	99	31.94		
VDR (c.2T>C; FokI)	TT	28	17.83	0.1284	>0.05
	TC	74	47.14		
	CC	55	35.03		
	T	130	41.40		
	C	184	58.60		
VDR (c.1024+283G>A; BsmlI)	AA	19	12.75	0.4746	>0.05
	GA	63	42.28		
	GG	67	44.97		
	A	101	33.89		
	G	197	66.11		

### Statistical Analysis

Statistical data was processed using the IBM Statistical Package for the Social Sciences, version 24. Arithmetic mean and standard deviation were used as measures to describe the initial sample, while the interpretation of the results obtained (which do not have a normal distribution) was carried out using median, as well as lower and upper quartiles: Q1 (25%) and Q3 (75%). When comparing averages or medians, the Mann–Whitney *U*-test was used. In the study of genetic polymorphism among groups, Fischer's exact test was also used. Differences were considered statistically significant at *p* < 0.05.

For genetic studies, we applied the parametric one-way analysis of variance (pairwise comparison with Scheffe test), non-parametric analysis of variance Kruskal–Wallis (pairwise comparison by Mann–Whitney), Chi-square test of independence for contingency table, and analysis of the proportion of children with various contents vitamin D with the help of *z*-test of equality of shares.

## Results

Children from 0–3 years of age had an average age of 1.92 ± 1.10 years; the median was 1.96 years (Q1–Q3= 1.00–2.77). The average age of children aged 4–10 years was 7.16 ± 2.02 years, and the median was 6.94 years (Q1–Q3= 5.52–8.72), while the average age of subjects aged 11–18 years was 13.85 ± 1.69 years, and the median was 13.59 years (Q1–Q3= 12.51–15.31).

The distribution of samples in the three age groups, depending on the prophylactic dose of cholecalciferol administered, is shown in Table 6. The number of subjects who did not receive prophylactic vitamin D was smallest at the age of 3 years; with age, the number of subjects who had vitamin D prophylaxis increased and was maximum among adolescents.

**Table 6:** The distribution of samples in the three study regions depending on prophylactic doses of cholecalciferol.

**Doses of cholecalciferol, IU**	**Studies numbers in Moscow, *n* (%)**	**Studies numbers in Krasnoyarsk, *n* (%)**	**Studies numbers in Stavropol, *n* (%)**	**Total, *n* (%)**
0	90 (59.6)	65 (47.4)	51 (32.9)	206 (46.4)
1–1,000	53 (35.1)	47 (34.3)	49 (31.0)	149 (33.4)
1,001–1,500	0 (0%)	16 (11.7)	45 (28.5)	61 (13.7)
≥1,500	8 (5.3)	9 (6.6)	12 (7.6)	29 (6.5)

The number of blood samples received from healthy children who took prophylactic dose of cholecalciferol >1,000 IU in Stavropol was 57 (36.1%), and in Moscow and Krasnoyarsk, these numbers were significantly lower−8 (5.3%) and 25 (17.3%), respectively (*p* < 0.05). About half of the subjects did not receive vitamin D prophylaxis (Table 7).

**Table 7:** The distribution of samples in the three age groups, depending on prophylactic doses of cholecalciferol.

**Doses of cholecalciferol, IU**	**Studies numbers in children 0–3 y.o., *n* (%)**	**Studies numbers in children 4–10 y.o., *n* (%)**	**Studies numbers in children 11–18 y.o., *n* (%)**
0	26 (24.3)	87 (44.8)	93 (64.8)
1–1,000	54 (50.5)	60 (30.9)	35 (24.1)
1,001–1,500	18 (16.8)	34 (17.5)	9 (6.2)
≥1,500	9 (8.4)	13 (6.7)	7 (4.8)

The results of the study showed that the average blood level of vitamin D in healthy subjects was 32.74 ± 17.47 ng/ml (median = 29.60, Q1–Q3 = 21.68–39.70). In general, a normal blood level of 25(OH)D was observed in 45.5% of cases, insufficiency in 30.1%, deficiency in 19.8%, and severe deficiency in 1.6%.

In a comparative analysis of the average level of 25(OH)D in children of different ages and sexes, significant difference was found between girls and boys in the older age group (*p* = 0.033). Adolescent boys had lower values of 25(OH)D, which was 25.70 ± 11.21 (median = 24.10, Q1–Q3= 17.40–30.51) ng/ml, compared with adolescent girls who had 28.70±14.01 (median = 26.18, Q1–Q3= 18.80–33.56) ng/ml, which allows them to be classified as a risk group at this age.

Calcidiol level reduced with increasing age: in healthy children, it decreased from 41.36 ng/ml at an early age to 26.94 ng/ml in adolescence (*p* < 0.001; Table 8). In addition, age patterns of vitamin D availability in the subjects were uniform—as a rule, from the maximum level reached in the first year of life, against the background of prophylactic administration of cholecalciferol, a progressive decrease occurred with increasing age, culminating in the development of vitamin D deficiency and insufficiency by school age.

**Table 8:** Average level of 25(OH)D (ng/ml) in children and adolescents of the three study regions in different seasons.

**Season**	**Level 25(OH)D, ng/ml M** **±** **m, Me (Q1–Q3)**				
	**Moscow, (M)**	**Krasnoyarsk, (K)**	**Stavropol, (S)**	**General group**	***p* between regions**
Winter (1)	31.71 ± 14.44 29.20 (22.90–39.20)	38.40 ± 29.41 29.45 (22.70– 39.95)	30.07 ± 12.56 31.70 (18.20–36.00)	33.38 ± 19.81 9.20 (22.90–39.10)	*p* > 0.05
Spring (2)	26.43 ± 26.42 15.05 (10.70–36.65)	23.17 ± 9.62 21.16 (15.47–28.54)	35.65 ± 19.62 32.00 (21.60–43.40)	28.66 ± 18.17 23.47 (16.40–34.10)	*p*M2-S2 = 0.031 *p*K2-S2 = 0.005
Summer (3)	39.02 ± 16.04 31.65 (26.80–48.54)	32.23 ± 14.56 29.16 (22.51–34.45)	35.16 ± 14.31 31.07 (24.57–44.56)	34.96 ± 14.93 30.70 (24.57–42.59)	p>0.05
Autumn (4)	34.68 ± 15.55 31.30 (24.00–40.80)	27.26 ± 22.11 21.24 (15.11–30.30)	34.29 ± 13.15 31.60 (24.10–43.91)	32.90 ± 16.28 30.30 (21.68–40.14)	*p*M4-K4 = 0.002 *p*S4-K4 = 0.000
Total	33.26 ± 16.35 30.10 (22.20–41.61)	30.50 ± 21.16 25.00 (19.47–35.30)	34.20 ± 14.65 31.60 (23.50–43.56)	32.74 ± 17.47 29.60 (21.68–39.70)	*p*M-K = 0.008 *p*K-S = 0.000
*p* between seasons	*p*M1-2 = 0.025 *p*M2-3 = 0.008 *p*M2-4 = 0.016	*p*K1-2 = 0.002 *p*K1-4 = 0.006 *p*K2-3 = 0.002 *p*K3-4 = 0.008	*p* > 0.05	*p*1-2 = 0.008 *p*2-3 = 0.000 *p*2-4 = 0.005	

*Mann–Whitney test was applied*.

**Figure 1:**
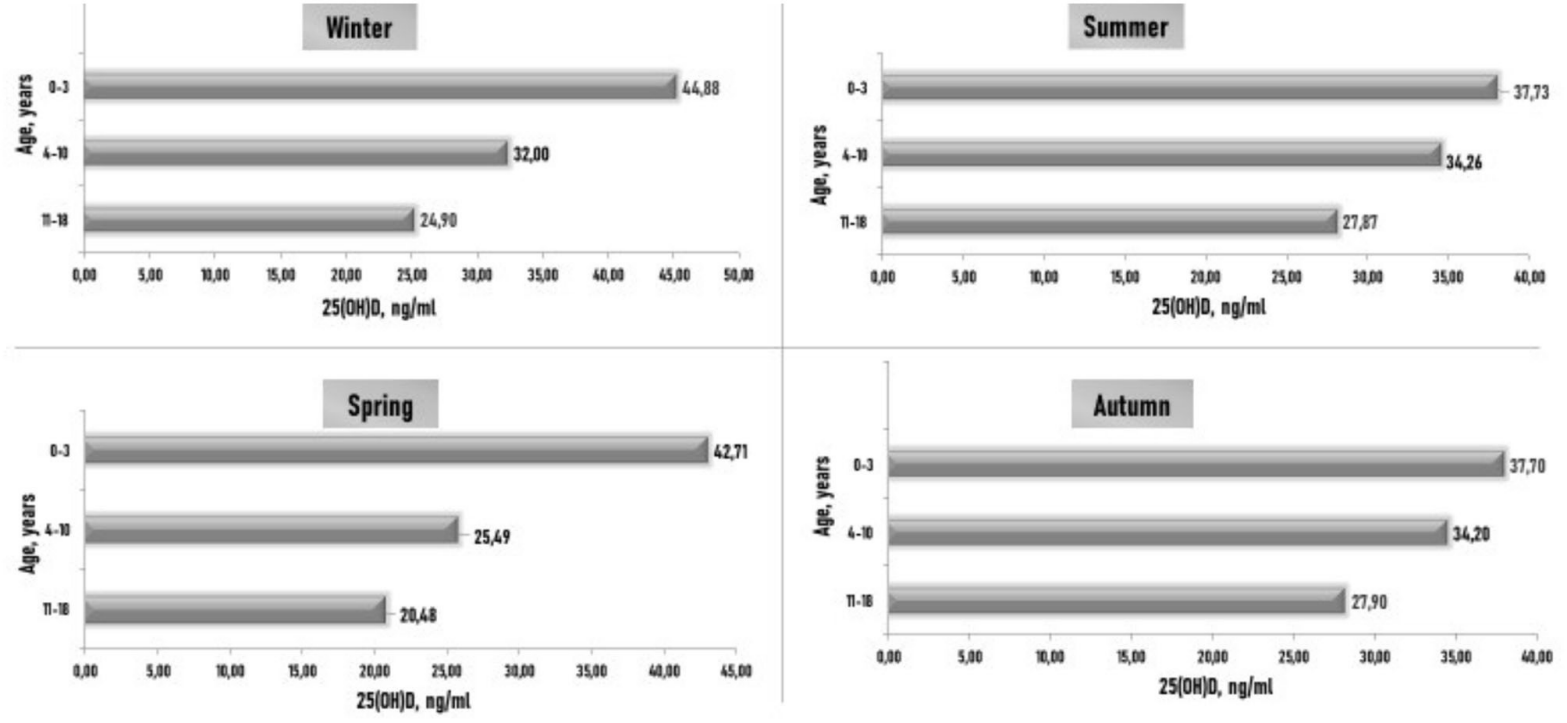
Subjects' blood 25(OH)D (ng/ml) concentrations in various age groups and different seasons. *p* is the level of significance of differences: p1–3 (1) < 0.05 is the significance between the level of 25(OH)D in winter and summer in children aged 0–3 years; p2–3 (2) < 0.05 is the significance between the levels of 25(OH)D in spring and in summer in children aged 4–10 years; p2–3 (3) < 0.05 is the significance between the levels of 25(OH)D in spring and in summer in children aged 11–18 years.

In healthy children from 0–3 years of age, the average level of 25(OH)D was normal in all seasons. In children aged 4–10 years, significantly lower levels of 25(OH)D were recorded in spring than in summer. The average level of 25(OH)D in older children was below normal in all seasons, and the lowest levels were observed in spring and winter in adolescents (Figure 1).

**Figure 2:**
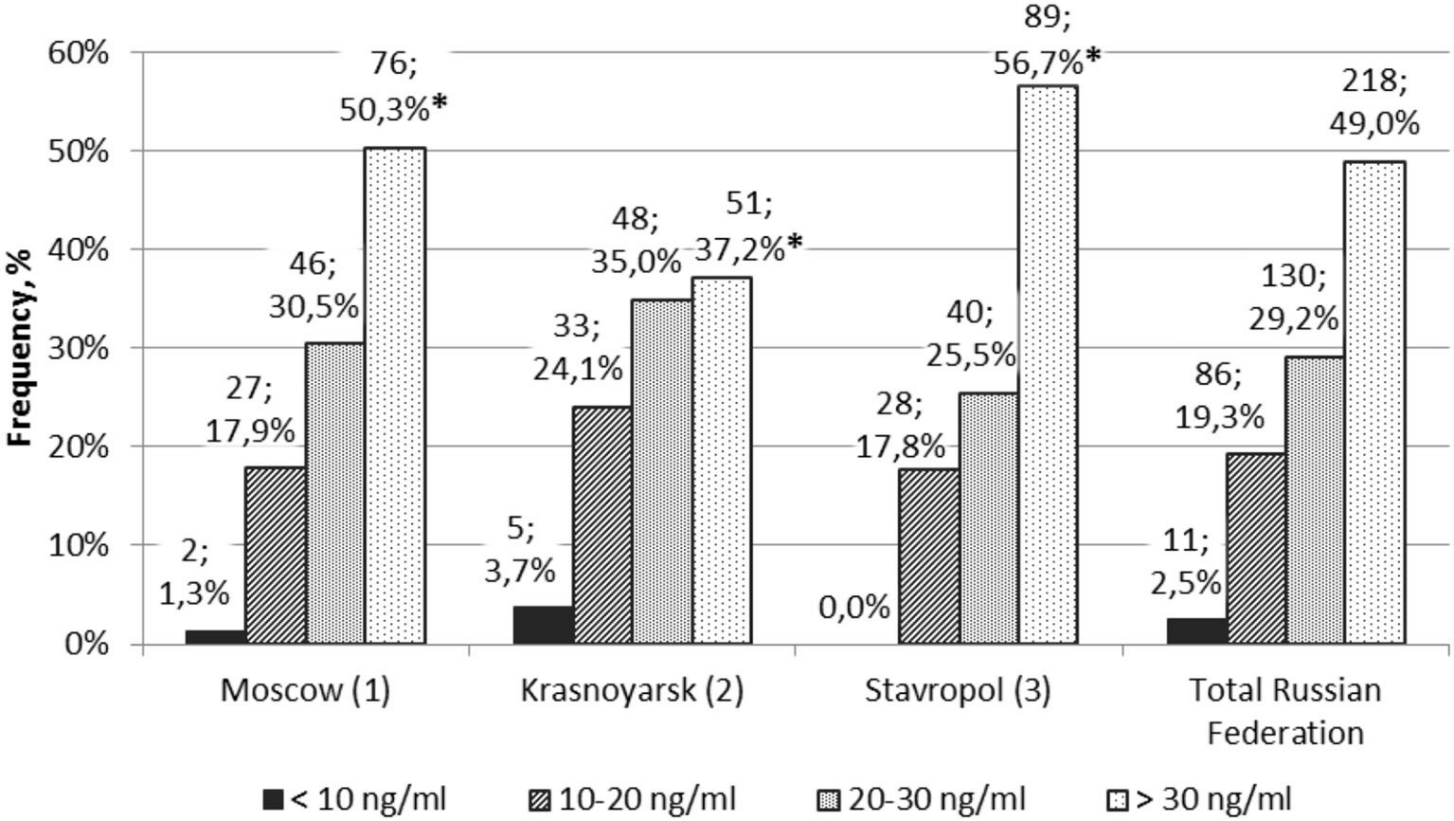
Frequency of healthy children and adolescents with different levels of vitamin D (*n*, %) in different regions. *p1–2 < 0.05 and p2–3 < 0.05 are Z criteria for comparing the proportions of children.

The blood levels of 25(OH)D in healthy subjects in different regions varied (Figure 2). The number of children with normal level of 25(OH)D was significantly higher in the Moscow region and the Stavropol territory than in the Krasnoyarsk territory (*p* < 0.05). There were no statistically significant differences in the number of subjects in the surveyed regions with respect to deficiency, insufficiency, and normal vitamin D availability.

The average serum levels of 25(OH)D (ng/ml) among subjects in different seasons in the three regions of the Russian Federation studied are shown in Table 8. Significant differences were observed when the values of 25(OH)D were compared among the regions in spring and autumn. This corresponds to a greater number of sunny days in the Stavropol territory compared to the Moscow region and the Krasnoyarsk territory. Thus, in spring, the DS was 742.2 h in the Moscow region, 561.3 h in the Krasnoyarsk territory, and 687 h in the Stavropol territory. DS in the summer was 883.8 h in the Moscow region, 896.6 h in the Krasnoyarsk territory, and 1,013.3 h in the Stavropol territory. In the fall, DS was 391.7 h in the Moscow region, 345.2 h in the Krasnoyarsk territory, and 559.3 h in the Stavropol territory.

In the Stavropol territory, there were no significant differences in the blood levels of 25(OH)D across the seasons, which is primarily due to the fact that subjects in the region more often took prophylactic doses of cholecalciferol than subjects in other regions. In the Moscow region, higher blood levels of 25(OH)D were observed in summer than in spring. In the Krasnoyarsk territory, subjects had the highest average 25(OH)D levels in winter, while the lowest levels were detected in spring. This may be due to greater commitment to preventing vitamin D deficiency in patients during winter. In addition, the DS during winter was 2 times higher (175.6 h) in the Krasnoyarsk territory than in other regions: 81.7 h in the Moscow region and 87.4 h in the Stavropol territory. When comparing the level of vitamin D in the general group of children, a significantly low value of 25(OH)D is also celebrated in the spring.

**Figure 3:**
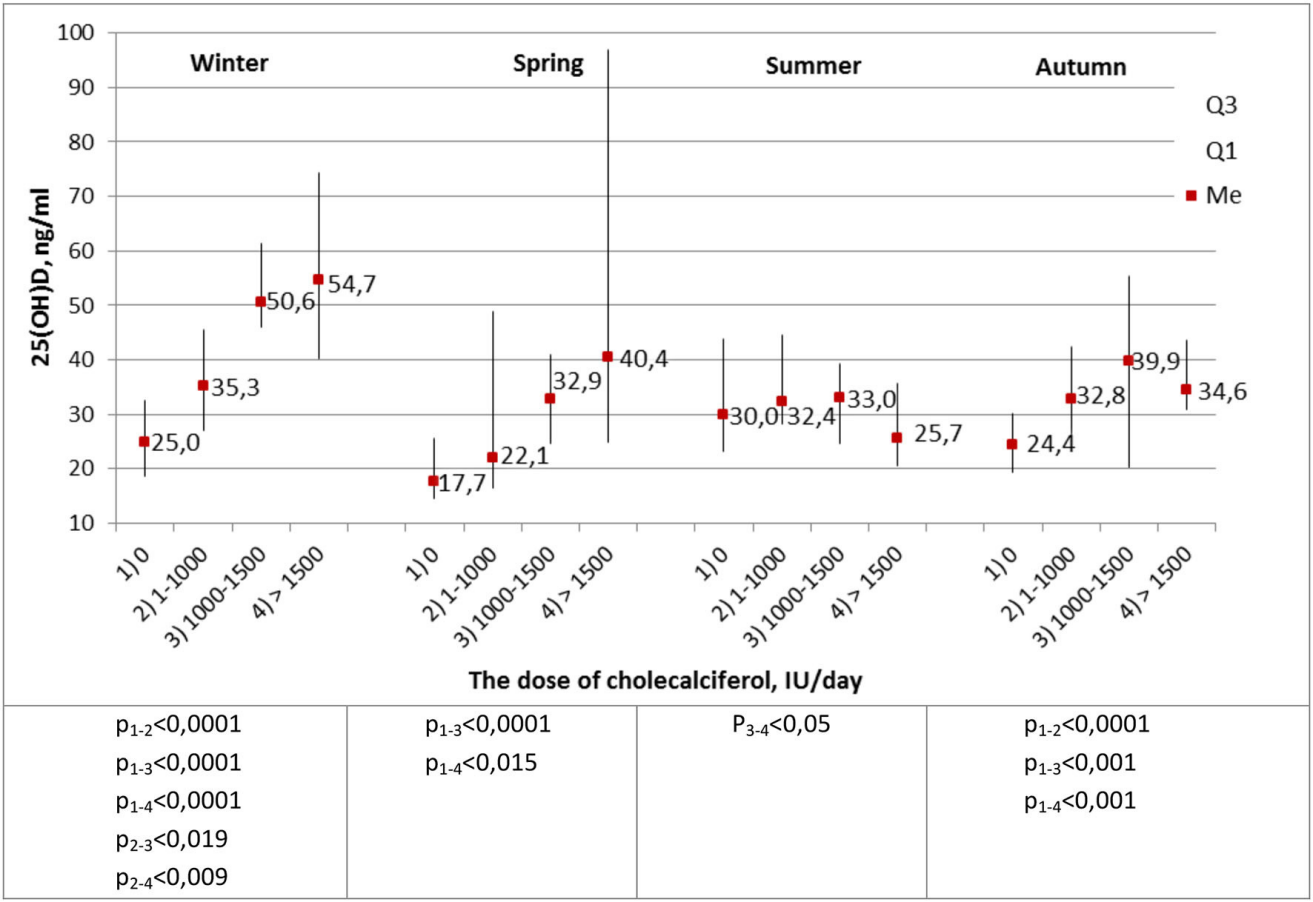
Average blood levels of 25(OH)D (ng/ml) in healthy children and adolescents, depending on the prophylactic dose of cholecalciferol. The Kruskal–Wallis criterion.

The most important stage of the analysis was the comparison of calcidiol levels in healthy subjects with respect to the daily prophylactic dose of cholecalciferol administered (Figure 3).

During winter, the level of 25(OH)D significantly increased depending on the prophylactic dose of cholecalciferol administered. In spring and autumn, significantly low concentrations of calcidiol were recorded in subjects who did not take prophylactic cholecalciferol. Thus, a dose of cholecalciferol ≥1,000 IU per day was enough to achieve normal blood level of 25(OH)D in healthy subjects, regardless of the season of the year.

The high level of 25(OH)D among subjects in the Stavropol territory can be explained by their high compliance with cholecalciferol prophylaxis, as some of them participated in studies on the prevalence of vitamin D deficiency and insufficiency in the Russian Federation (Rodnichok, Rodnichok 2) (21, 22).

The relationship between blood level of 25(OH)D in healthy subjects and variants of CYP2C9^*^3 (c.1075A>C; I359L, rs1057910), CYP3A4^*^3 (c.1334T>C; M445T, rs4986910), CYP2C9^*^2 (c.430C>T; R144C, rs1799853), CYP2D6^*^4 (1846G>A, rs3892097), and CYP3A4^*^1B (c.−392C>T, rs2740574) was studied in winter and spring due to low calcidiol levels in these seasons of the year. However, no relationships were established (Table 9).

**Table 9:** Average level of 25(OH)D (ng/ml) in subjects in winter, depending on the genotypes of cytochrome gene polymorphisms.

**Allele**		***n***	**Level 25(OH)D in winter, M ± m, Me (Q1-Q3)**	***p***
CYP2C9*3 (1075A>C; I359L)	AA CA	106 14	35.3 ± 20.6, 31.7 (23.5–44.6) 31.7 ± 21.3, 25.0 (15.0–35.9)	0.241
CYP3A4*3 (c.1334T>C; M445T)	TT CT	84 0	34.9 ± 20.6, 31.5 (23.2–41.8) -	–
CYP2C9*2 (c.430C>T; R144C)	CC CT	98 22	34.8 ± 21.6, 31.7 (23.1–39.2) 35.6 ± 15.9, 28.6 (23.5–45.0)	0.618
CYP2D6*4 (c.1846G>A)	AA AG GG	7 31 79	25.6 ± 11.2, 26.7 (13.3–31.3) 43.7 ± 32.4, 32.7 (23.6–47.5) 32.1 ± 13.4, 31.7 (22.3–39.7)	0.119
CYP3A4 *1B (c.-392C>T)	TT CT	113 6	35.1 ± 20.5, 31.7 (23.3–40.8) 25.0 ± 14.4, 23.1 (11.6–36.0)	–

*p is the significance level of differences, and Kruskal–Wallis test was applied*.

Molecular effects, including effects on levels of hormones, growth and inflammation factors, proteins, and, of course, blood calcium levels, are manifested through Vitamin D receptors (23–25).

It was found that the TT and TC genotypes of the c.1056T>C (TaqI) polymorphism were more common (~4-fold) in the Russian population than the CC genotype (Table 10). The frequency of the C allele was 2 times lower than that of the T allele. For the c.2T>C (FokI) polymorphism, children with CC and TC genotypes predominated. The frequency of homozygous TT genotype in children was lower (17.83%) than the CC and TC genotypes. In relation to the polymorphic variant c.1024+283G>A (BsmI), GG and GA genotypes occurred more frequently than AA homozygotes (12.75%). The frequency of the A allele was 2 times lower than that of the G allele.

**Table 10:** Frequency of alleles and genotypes of the *VDR* gene in a group of children from Russia.

**Genotype/allele**	**Children**		Russian^*^		French^**^	Japan^***^
	***N***	**%**	***N***	**%**	**%**	**%**
TaqI	155		138		189	488
TT	74	47.74	63	45.7	33.0	77.0
TC	63	40.65	60	43.5	49.0	22.0
CC	18	11.61	15	10.9	18.0	1.0
T	211	68.06	186	67.4	57.0	88.0
C	99	31.94	90	32.6	43.0	12.0
FokI	157		96		100	249
TT	28	17.83	13	13.5	10.0	12.0
TC	74	47.14	55	57.3	47.0	51.0
CC	55	35.03	28	29.2	43.0	37.0
T	130	41.40	81	42.2	33.5	37.5
C	184	58.60	111	57.8	66.5	62.5
BsmlI	149		96		189	102
AA	19	12.75	7	7.3	17.0	7.0
GA	63	42.28	40	41.7	51.0	38.0
GG	67	44.97	49	51.0	32.0	55.0
A	101	33.89	54	28.1	42.3	26.0
G	197	66.11	138	71.9	57.7	74.0

* *(25, 26)*,

** *(27)*,

*** *(28)*.

## Discussion

The purpose of this study was to analyze the influence of exogenous factors on the blood levels of 25(OH)D in children of three regions of the Russian Federation, as well as the relationship of blood 25(OH)D levels with polymorphic variants of cytochrome P450 genes and *VDR* gene. The problem of vitamin D deficiency is currently relevant due to its widespread prevalence among both adults and children, including those in the Russian Federation. In this study, it was found that, in children, the level of 25(OH)D in the serum reduced with increasing age, and in adolescents, vitamin D deficiency was observed in all seasons of the year, with the lowest levels of 25(OH)D recorded in spring and winter.

A 2016 study of vitamin D deficiency in Europe also suggests that adolescents, on average, have a higher risk of vitamin D deficiency than the other age groups (26). In an American study on a sample of children from 1 to 11 years of age, the results showed lower levels of 25(OH)D in children aged 6–11 years (27). Russian studies have also shown better vitamin D availability in young children compared to adolescents (28, 29). It is obvious that age-related patterns of vitamin D availability in children of the Russian Federation depend on prophylactic administration of cholecalciferol at an early age, which is achieved by ongoing programs (the National Program for Optimizing Vitamin and Mineral Availability in Children in Russia, and the national program “vitamin D deficiency in children and adolescents in the Russian Federation: modern approaches to correction” (20). However, the recommendations of these programs are not followed in older children, and there is a progressive decrease in calcidiol levels, culminating in the development of vitamin D deficiency and insufficiency by school age.

Taking into account the peculiarities of vitamin D synthesis and the geographical location of most parts of the Russian Federation in the northern hemisphere above the 42nd parallel (30), the majority of children do not have optimal vitamin D levels in winter and spring (31).

The results of this study revealed that the region of residence also affects the availability of vitamin D. The regions that were studied are located at different geographical latitudes: Moscow at 55° s. l., Krasnoyarsk at 56° s. l., and Stavropol at 45° s. l. and, they have different numbers of sunny days per year and in different seasons of the year. The lowest DS during the year was recorded in the Krasnoyarsk territory, and this explains the low percentage of children with normal levels of 25(OH)D in the province.

The average level of 25(OH)D in adolescent boys was statistically significantly lower than that in adolescent girls. According to recent data, there are publications about similar conclusions among young adolescences (32).

We also found that the level of 25(OH)D significantly depended on the prophylactic dose of cholecalciferol administered. A dose of cholecalciferol ≥1,000 IU per day could achieve normal level of 25(OH)D in healthy children. This is consistent with the data of the national program (the National Program for Optimizing Vitamin and Mineral Availability in Children in Russia, and the national program “vitamin D deficiency in children and adolescents in the Russian Federation: modern approaches to correction”), which suggests a scheme for preventing hypovitaminosis D in children of the Russian Federation by administering 1,000–1,500 IU per day of cholecalciferol (20).

We therefore studied the frequency of alleles and genotypes of the *VDR* gene among the study subjects. The results obtained, presented in Table 9, completely coincide with the distribution of genotypes in the adult Russian population and partially coincide with the genotypes in populations of France and Japan (33–36).

We found no statistically significant association between single-nucleotide polymorphic variants of cytochrome P450 genes (CYP2C9^*^3, CYP3A4^*^3, CYP2C9^*^2, CYP2D6^*^4, and CYP3A4^*^1B) and blood level of 25(OH)D in the study subjects. As noted above, the enzymes encoded by these genes are involved in the process of hydroxylation, but they do not affect the content of serum vitamin D. In a 2016 review, Jolliffe et al. analyzed the association of 11 gene polymorphisms (*DHCR7, CYP2R1, CYP3A4, CYP27A1, DBP, LRP2, CUB, CYP27B1, CYP24A1, VDR*, and *RXRA*) with extracostal effects and circulating 25(OH) levels. In that study, statistically significant associations were found for 55 polymorphic variants in the 11 studied genes (37).

Based on the results of the present study, the distribution of frequencies of alleles of the *VDR* gene was studied (Table 10). Similar data were obtained by Kozlov et al. who studied the frequency of *VDR* genotypes by FokI and BsmI alleles and found that the Russian population had more carriers of the TT genotype than the CC genotype of TaqI polymorphism (28 and 13%, respectively) and that the GG genotype was predominant compared to the AA genotype of Bsml polymorphism (49 and 7%, respectively) (38). Baroncelli et al. studied 209 healthy children and revealed a 2-fold predominance of the G allele of BsmI polymorphism compared to the A allele (39). In a study of 202 children in Saudi Arabia, similar results were obtained (40).

Analysis of the level of 25(OH)D in the subjects did not reveal dependence on genotypes of the three polymorphic variants of the VDR gene (Table 11), which coincides with data from international studies (41). For example, in a study of 642 healthy children in Denmark, VDR gene polymorphism was not proven to have any effect on vitamin D levels (42). Similar results were also obtained in a survey of 222 adults in the UK (43). It can therefore be assumed that polymorphic variants do not affect the serum level of vitamin D, but can affect its function by changing its binding sites. In addition, a change in the carrier of the FokI polymorphic variant leads to the formation of a short form of the VDR protein, which leads to higher transcriptional activity and increased cytokine expression, and may be associated with autoimmune and infectious diseases (44).

**Table 11:** Average level of 25(OH)D (ng/ml) in subjects depending on the genotypes of *VDR* gene polymorphisms.

***VDR*** **polymorphism**		***N***	**M±m 25(OH)D (HT/MJI)**	***p***
c.1056T>C(A>G) TaqI	TT	74	33.7 ± 18.2	0.857
	TC	63	32.8 ± 22.5	
	CC	18	28.3 ± 10.3	
	Total	155	32.7 ± 19.6	
c.2T>C FokI	TT	28	30.5 ± 20.5	0.453
	TC	74	33.0 ± 20.9	
	CC	55	32.8 ± 19.3	
	Total	157	32.5 ± 19.3	
c.1024+283G>A BsmlI	AA	19	29.2 ± 10.2	0.858
	GA	63	32.8 ± 22.8	
	GG	67	33.6 ± 17.5	
	Total	149	32.7 ± 19.2	

*p is the level of significance of differences, and Kruskal–Wallis test was applied*.

The following conclusions can be drawn from this study. The subjects that were most at risk for vitamin D deficiency among healthy subjects in the study sample from the three studied regions of Russia were adolescents, especially males. In winter and spring, the lowest levels of 25(OH)D were recorded, regardless of the region of residence. Children of the Moscow region and the Stavropol territory had higher serum levels of 25(OH)D than children of the Krasnoyarsk territory. A prophylactic dose of ≥1,000 IU cholecalciferol per day can achieve normal level of 25(OH)D in healthy children.

However, there were limitations to this study. Samples used to evaluate the vitamin D levels were not taken from the same subject during different seasons of the year. The inclusion of new patients allowed us to evaluate prevention strategies for vitamin D deficiency in children during different seasons of the year. This study determined the duration of sunshine in the regions, not the children's exposure to the sun.

Therefore, for healthy children of the Russian Federation, exogenous factors such as time of the year, place of residence, and preventive administration of cholecalciferol, as well as intrinsic factors such as age and sex, play a determining role in the development of vitamin D deficiency; while genetic factors—polymorphic variants of the genes of phase 1 enzymes of xenobiotic metabolism (*CYP2C9, CYP2C19, CYP2D6, CYP3A4*) and the *VDR* gene—do not. A diagnostic algorithm incorporating age, season of the year, and prophylactic dose of cholecalciferol to identify patients at risk for vitamin D deficiency will be beneficial for the Russian population.

## Data Availability Statement

The raw data supporting the conclusions of this article will be made available by the authors, without undue reservation.

## Ethics Statement

The studies involving human participants were reviewed and approved by EC of the Research Center for Medical Genetics. Written informed consent to participate in this study was provided by the participants' legal guardian/next of kin.

## Author Contributions

EK and SK performed the conceptualization and provided the proof outline for the research. IZ contributed to the original idea. NI and LK supervised the research. NP, AZ, EZ, VC, SD, AV, VS, YM, RB, and VK all carried out the research. EK wrote the manuscript with support from AZ, EZ, and EL. All authors discussed the results and contributed to the final manuscript.

## Conflict of Interest

The authors declare that the research was conducted in the absence of any commercial or financial relationships that could be construed as a potential conflict of interest.

## Abbreviations

DS: duration of sunshine
VDR: vitamin D receptor.
